# “I Think You Are Trustworthy, Need I Say More?” The Factor Structure and Practicalities of Trustworthiness Assessment

**DOI:** 10.3389/fpsyg.2022.797443

**Published:** 2022-04-01

**Authors:** Michael A. Lee, Gene M. Alarcon, August Capiola

**Affiliations:** ^1^General Dynamics Information Technology, Inc., Dayton, OH, United States; ^2^Airman Systems Directorate, Air Force Research Laboratory, Wright-Patterson Air Force Base, Dayton, OH, United States

**Keywords:** trustworthiness, structural equation modeling, bifactor analysis, organizational outcomes, hierarchical regression

## Abstract

Two popular models of trustworthiness have garnered support over the years. One has postulated three aspects of trustworthiness as state-based antecedents to trust. Another has been interpreted to comprise two aspects of trustworthiness. Empirical data shows support for both models, and debate remains as to the theoretical and practical reasons researchers may adopt one model over the other. The present research aimed to consider this debate by investigating the factor structure of trustworthiness. Taking items from two scales commonly employed to assess trustworthiness, we leveraged structural equation modeling to explore which theoretical model is supported by the data in an organizational trust context. We considered an array of first-order, second-order, and bifactor models. The best-fitting model was a bifactor model comprising one general trustworthiness factor and ability, benevolence, and integrity grouping factors. This model was determined to be essentially unidimensional, though this is qualified by the finding that the grouping variables accounted for significant variance with for several organizational outcome criteria. These results suggest that respondents typically employ a general factor when responding to items assessing trustworthiness, and researchers may be better served treating the construct as unidimensional or engaging in scale parceling of their models to reflect this response tendency more accurately. However, the substantial variance accounted by the grouping variables in hierarchical regression suggest there may be contexts in which it would be acceptable to consider the theoretical factors of ability, benevolence, and integrity independent of general trustworthiness.

## Introduction

Interpersonal trust is a fundamental component of relationships established both within the workplace (Colquitt et al., 2007) and in daily life (Luchies et al., 2013). In their seminal paper, Mayer et al. (1995) separated one’s willingness to be vulnerable to others and engagement in that vulnerability (i.e., trust and risk-taking in the relationship, respectively) from the perception of others as willing or able to commit to their promises (i.e., trustworthiness). While this work was initially rooted within the context of trust within organizations, researchers have since applied this model to a range of contexts, including general interpersonal trust (Lewicki et al., 2006), consumer trust in e-commerce (Jarvenpaa et al., 2000), and trust in automation (Lee and See, 2004). Mayer and colleagues’ conceptualization delineates the trustworthiness construct into three interrelated factors: ability, benevolence, and integrity (see also Schoorman et al., 2007, 2016). Prior meta-analytic research has shown large, positive relationships between the three factors of trustworthiness, trust, and various workplace outcomes (e.g., citizenship behaviors; see Colquitt et al., 2007).

Despite the prevalence of Mayer et al.’s (1995) theoretical model, empirical research on the factor structure of trustworthiness remains ongoing and at times, contested. Indeed, other researchers have proposed a two-factor model of trust, comprising cognition- and affect-based trust (McAllister, 1995). This model has been interpreted as being the result of unique trustworthiness perceptions (see Colquitt et al., 2007), and meta-analytic research has also leveraged this factor structure (Dirks and Ferrin, 2002; De Jong et al., 2016). Unlike the theoretical model proposed by Mayer et al. (1995), the McAllister (1995) model does not delineate between trustworthiness and trust, but rather emphasizes the distinct cognition- and affect-based psychological processes that occur over the course of trust formation. As a result, some researchers have interpreted that the scale created by McAllister (1995) as one that measures trustworthiness through the cognition- and affect-based lenses (Dietz and Hartog, 2006; McEvily and Tortoriello, 2011). We now review the literature surrounding these contrasting trust models, as well as recent findings on the theoretical structure of trust.

Mayer et al. (1995) described a theoretical model of the trust process comprising various components. In early interactions, people often use general tendencies (i.e., dispositional trust) to determine others’ trustworthiness. As the trustor gathers information about the referent, the effects of dispositional trust decrease over time (Alarcon et al., 2016; Jones and Shah, 2016). Mayer et al. (1995) argued that three interrelated factors—ability, benevolence, and integrity—compose a trustor’s perception of a trustees’ trustworthiness. Perceived *ability* was defined as the perception that the trustee can perform one or more specific tasks. Perceived *benevolence* was described as the extent to which the trustee is perceived to have the trustor’s best interest in mind. Perceived *integrity* was defined as the extent to which the trustee values are perceived to be in alignment with the trustor’s values. *Trust* was defined as the extent to which the trustor is willing to be vulnerable to the trustee without being able to directly monitor their actions. Finally, actuating this willingness was defined as *risk-taking in the relationship* (alternatively, behavioral trust or trust action). Although research and theory indicate general tendencies, trust, and risk-taking in the relationship are important constructs, they are beyond the scope of the current study, which focuses on the theoretical composition and assessment of trustworthiness. Later empirical research demonstrated perceived ability, benevolence, and integrity have strong, positive intercorrelations with each other (Mayer and Davis, 1999; Colquitt et al., 2007; Poon, 2013). In these studies, the intercorrelations of ability, benevolence, and integrity were so large that it appears participants may have difficulty separating these factors in practice. For example, Colquitt et al. (2007) found meta-analytic correlations for ability, benevolence, and integrity were all above 0.60, with benevolence and integrity correlated at 0.68, corrected for range restriction and predictor reliability.

In terms of criterion-related validity, these trustworthiness factors have shown some differential relationships with relevant workplace outcomes. Colquitt et al. (2007) found that perceived ability had a smaller corrected meta-analytic correlation with counterproductive behaviors compared to perceived benevolence and integrity. However, these three factors rarely showed unique relationships with workplace outcomes simultaneously. Rather, at least two factors related similarly to workplace outcomes. For instance, the corrected meta-analytic correlations between both perceived benevolence and integrity with counterproductive behaviors were identical. Additionally, benevolence and integrity had nearly identical corrected meta-analytic correlations with citizenship behavior. Furthermore, Colquitt et al. explicitly noted that although the theoretical rationale for the three factors may be justified, participants may have difficulty distinguishing between the self-report items measuring ability, benevolence, and integrity. Similar findings for the trustworthiness factors have been found in virtual teams (Jarvenpaa et al., 1998; Aubert and Kelsey, 2003). Finally, the extent to which ability, benevolence, and integrity have shown hierarchical predictive validity above and beyond the effects of each other on relevant outcomes has received limited empirical attention (for an exception, see Colquitt et al., 2011).

Alternatively, the other popular theoretical conceptualization of trust is a two-factor model. McAllister (1995) proposed two separate forms of trust: cognition-based and affect-based trust. *Cognition-based trust* concerns perceptions of the referent’s dependability and reliability. *Affect-based trust* concerns empathy and emotional bonds between a trustor and a trustee, and thus requires more interaction between the two actors for it to develop. Therefore, cognition-based trust emerges before affect-based trust. However, once affect-based trust is established, it may supersede cognition-based trust in terms of its importance in maintaining a relationship (p. 30). In his structural model, McAllister demonstrated that both forms of trust were related, and that affect-based trust was positively associated with actor interaction frequency.

Some researchers have adopted McAllister’s (1995) two-factor model of trust, explicating the attributes which lead to trust and how those attributes overlap with the trustworthiness factors Mayer et al. (1995) proposed (e.g., Dirks and Ferrin, 2002; Colquitt et al., 2007, 2011; Coovert et al., 2017). In a review of measures of trust in organizational literature, McEvily and Tortoriello (2011) explicitly conclude that if one were to assess trustworthiness beliefs, measures developed by both McAllister (1995) and Mayer and Davis (1999) are viable options. It is thus no surprise that through leveraging Mayer et al.’s (1995) nomenclature, researchers have described cognition- and affect-based forms of trust*worthiness* as antecedents to one’s willingness to be vulnerable to another (e.g., Capiola et al., 2020). For instance, Colquitt et al. (2011) classified ability and integrity as cognition-based trustworthiness perceptions and benevolence as an affect-based trustworthiness perception when they investigated whether these were related to trust in contexts comprising differential predictability. Their findings showed evidence that cognition-based antecedents (ability and integrity) were more predictive of trust in unpredictable contexts, and benevolence was more predictive of trust in predictable contexts. However, separating ability from integrity led to a non-significant relationship between ability and trust in the unpredictable contexts, and integrity mirrored the relationship between benevolence and trust in predictable contexts. Thus, it appears there is intercorrelation between trustworthiness factors, regardless of how they are discretized. This parallels the opacity in both Colquitt et al.’s (2007) meta-analysis resulting in intercorrelations between ability, benevolence, and integrity, as well as De Jong et al.’s (2016) meta-analysis of team trust, which showed (perceiving a team as having attributes which led to) cognition- and affect-based trust correlated at 0.76.^1^ Taken together, there appears to be some muddiness in how trustworthiness is constructed, and one simplifying approach might be to measure trustworthiness as a single factor, regardless of one’s preferred model.

Tomlinson et al. (2020) recently tested a model which separated trustworthiness from trust by combining Mayer et al. (1995) and McAllister’s (1995) models. Importantly, Tomlinson et al. (2020) noted that McAllister (1995) did not differentiate between trust and trustworthiness like Mayer et al. (1995). As such, they argue McAllister’s (1995) model (or at least their “widely used” scale) is complicated by the fact that trust and trustworthiness are conflated (Tomlinson et al., 2020, p. 541). Tomlinson et al. demonstrated separating trust into cognition- and affect-based trust was a viable option and regressing those outcomes onto measures of trustworthiness would integrate both Mayer et al.’s (1995) and McAllister’s (1995) models. However, Tomlinson et al. (2020) did not systematically test the structure of trustworthiness per Mayer et al.’s model. Instead, they assessed trustworthiness with a measure of perceived ability, behavioral integrity (i.e., alignment between word and action), benevolence, and values congruence (i.e., congruence between the trustor’s and trustee’s values/principles). Then, they assessed cognition- and affect-based trust with a scale (i.e., Gillespie, 2003) that did not conflate trust and trustworthiness.^2^ Results showed that ability and behavioral integrity were more important for predicting cognition-based trust than values congruence (but not benevolence), and benevolence (but not values congruence) was consistently more important for predicting affect-based trust than ability.

With the results of Tomlinson et al. (2020) in mind, it remains to be seen if trustworthiness can be further simplified into cognitive and affective dimensions, as previous research (e.g., Colquitt et al., 2007; Capiola et al., 2020) has proposed, or if the three-factor structure proposed by Mayer et al. (1995) is a better fit. Though researchers leveraging the two-factor solution have showed they do predict relevant criteria (McAllister, 1995; Coovert et al., 2017), it is not clear whether trustworthiness more accurately (and practically) comprises ability, benevolence, and integrity dimensions or cognition- and affect-based dimensions. In the present research, we aim to test structural models of trustworthiness to determine whether it best comprises a two- or three-factor solution, or if trustworthiness as a single factor might be the most practical and accurate representation of the construct. Given the high intercorrelations between ability, benevolence, and integrity (Colquitt et al., 2007), as well as cognition- and affect-based factors of trustworthiness (De Jong et al., 2016), the question remains: are we justified in delineating our measurement and analyses of trustworthiness? Both accurate and practical measurement are key in assessing psychological constructs, particularly in the workplace where it may be less than desirable for employees or other respondents to slog through more self-report measures than necessary. Such practicality of a single-factor solution might lead researchers to adopt a single-item measure, particularly if the item can reference an unambiguous construct (e.g., Wanous et al., 1997).

For the current study, we examined the factor structure of items from both the Mayer and Davis (1999) and McAllister (1995) scales to ensure the models are not unique to their corresponding scales. To this day, these scales have served as two of the most predominantly employed measures of trustworthiness in literature, but the research examining their underlying factor validity has been surprisingly scant. At the time of this writing, both scales have been cited thousands of times (based on a Google Scholar search), as well as employed in research published as recently as the same month of this writing (e.g., Navarro-Picado et al., 2022; Tacke et al., 2022). In many cases, the scales are not modified beyond changing the referent, or only items representing some of the factors are used. Despite this apparent interest and perceived utility, there have been few major efforts to validate or update these scales, nor to develop new scales for these factor structures. One recent attempt to create an updated scale using the Mayer et al. (1995) factors is the Muenster Epistemic Trustworthiness Inventory (METI, Hendricks et al., 2015), but that was specifically designed to assess people’s trust in experts and does not carry as wide an application as the Mayer and Davis (1999) scale. As both Mayer and Davis’ and McAllister’s (1995) scales (and their respective theoretical models) were initially developed within the organizational trust context, we chose to examine their factor structures specifically within the context of the employee-supervisor relationship, with employees serving as respondents. Based on this, we sought to answer the following research question:

*Research Question 1*: Which model structure best fits the underlying factor structure of trustworthiness?

**Figure 1:**
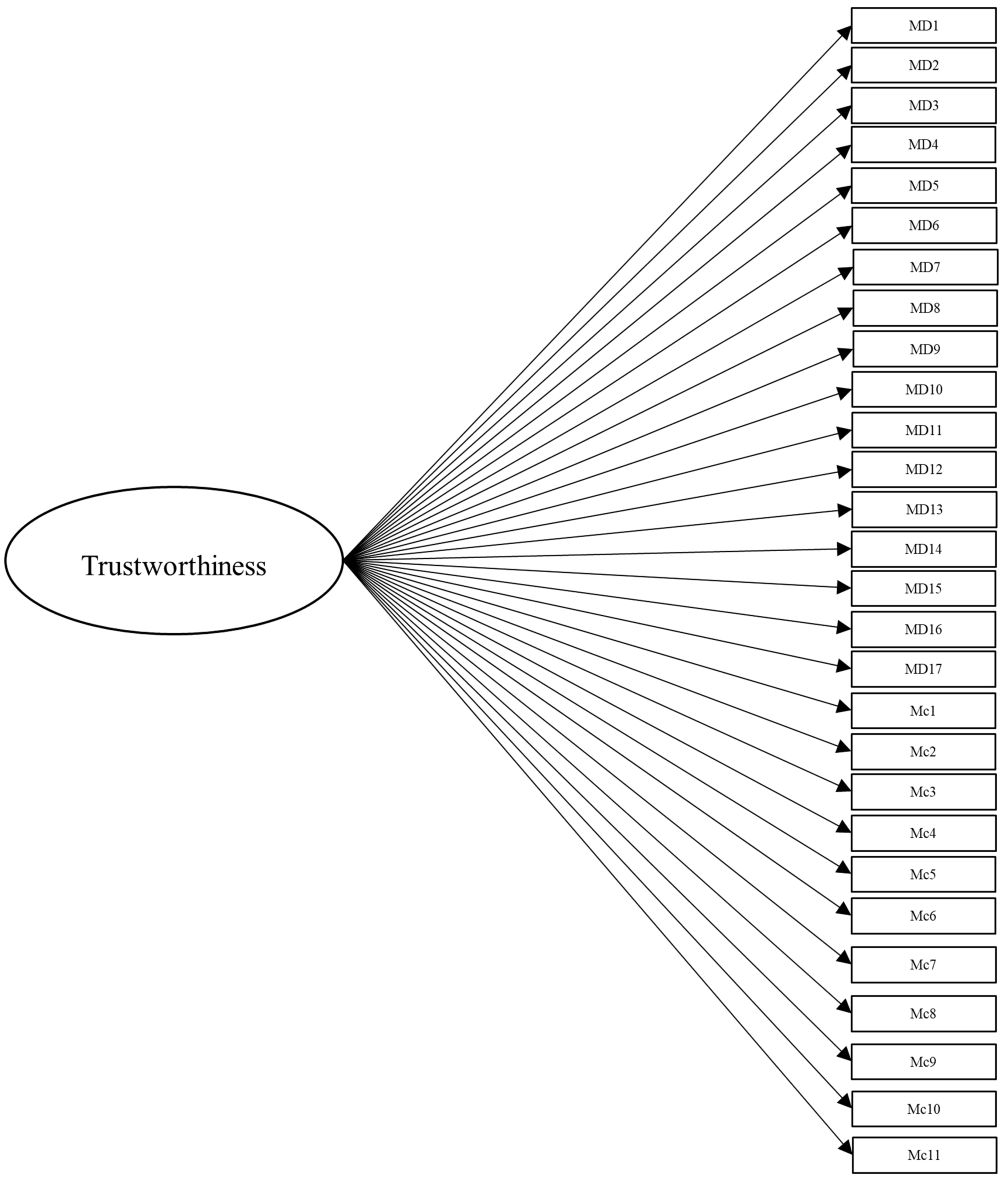
Unidimensional model with one general trustworthiness factor comprising 28 items from Mayer and Davis (1999) and McAllister (1995) combined. MD, Mayer and Davis (1999) items. Mc, McAllister (1995) items.

**Figure 2:**
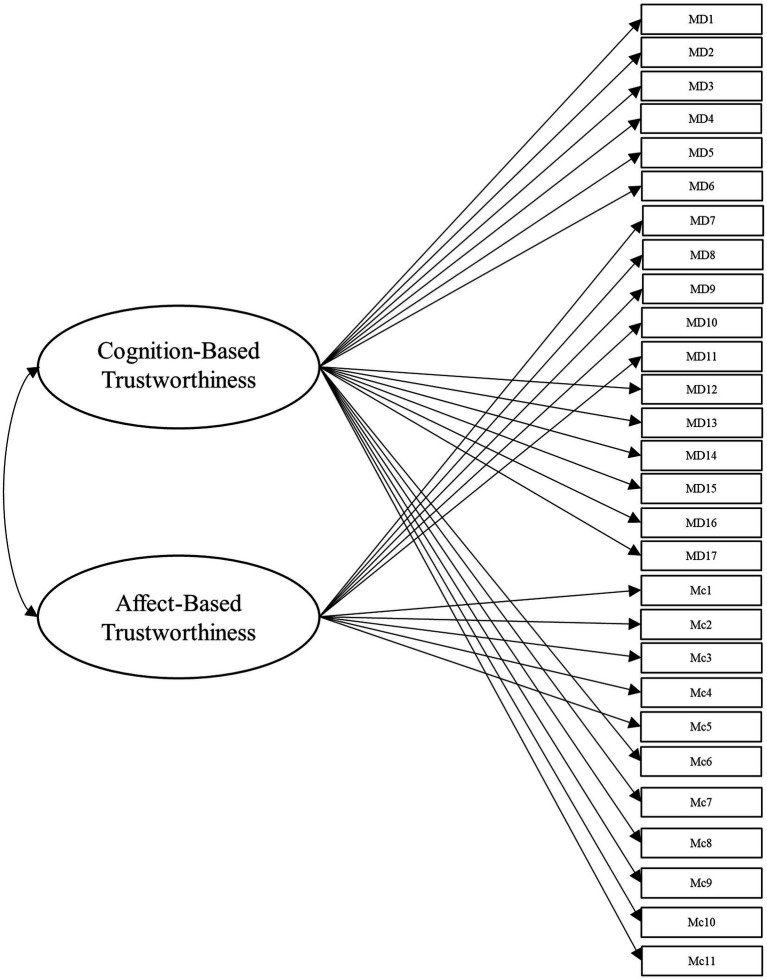
Two-dimensional model with cognition- and affect-based trustworthiness latent factors. The cognition-based trustworthiness factor comprises ability and integrity items from Mayer and Davis (1999) as well as McAllister’s (1995) cognition-based trust items, while the affect-based trustworthiness factor comprises benevolence items from Mayer and Davis (1999) as well as McAllister’s (1995) affect-based items. MD, Mayer and Davis (1999) items. Mc, McAllister (1995) items.

**Figure 3:**
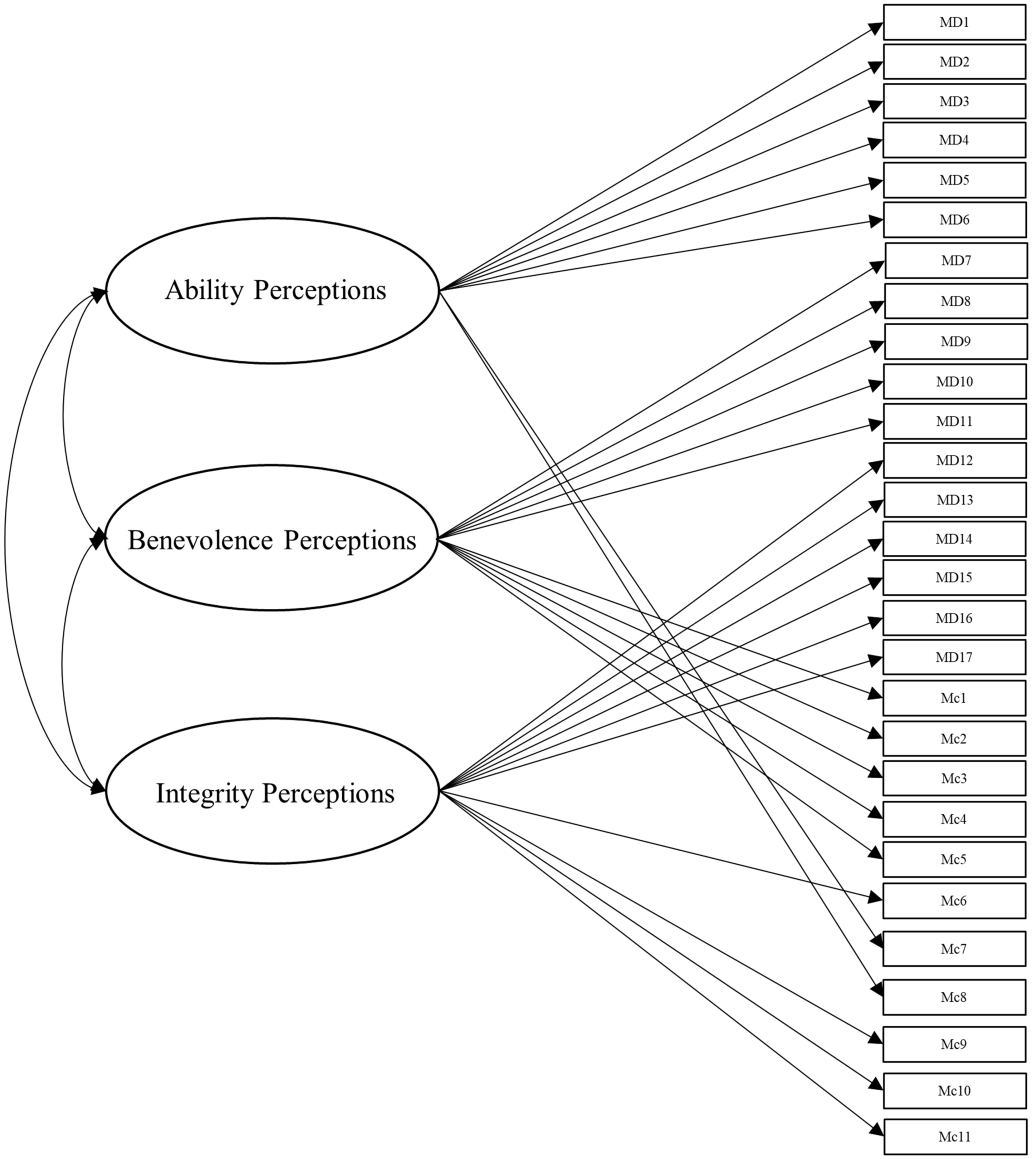
Three-dimensional model with ability, benevolence, and integrity latent factors correlated. All three factors comprised the corresponding items from Mayer and Davis’ (1999) scale as well as McAllister’s (1995) scale. In regard to the latter, etc., the ability and integrity factors in part comprised the items from McAllister’s (1995) scale classified into the corresponding categories by the research team (see Data Analysis), and the benevolence factor comprised the affect-based items from McAllister’s scale. MD, Mayer and Davis (1999) items. Mc, McAllister (1995) items.

**Figure 4:**
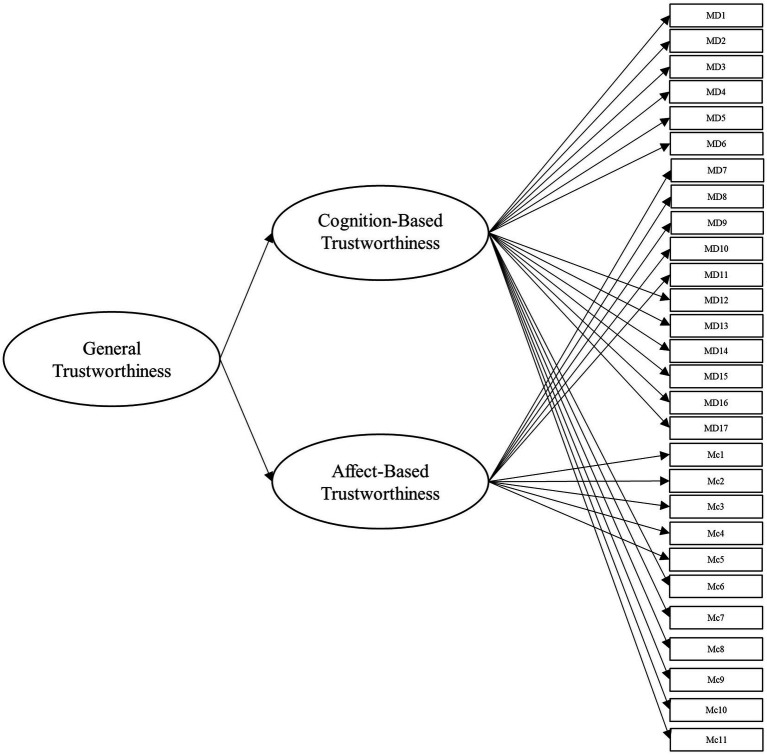
Second-order model with general trustworthiness latent factor. The first-order factors represent cognition- and affect-based trustworthiness. The cognition-based trust latent factor comprises ability and integrity items from Mayer and Davis (1999) as well as cognition-based trust items from McAllister (1995). The affect-based trustworthiness latent factor comprises Mayer et al.’s (1999) benevolence items as well as affect-based trust items from McAllister (1995) combined. MD, Mayer and Davis (1999) items. Mc, McAllister (1995) items.

**Figure 5:**
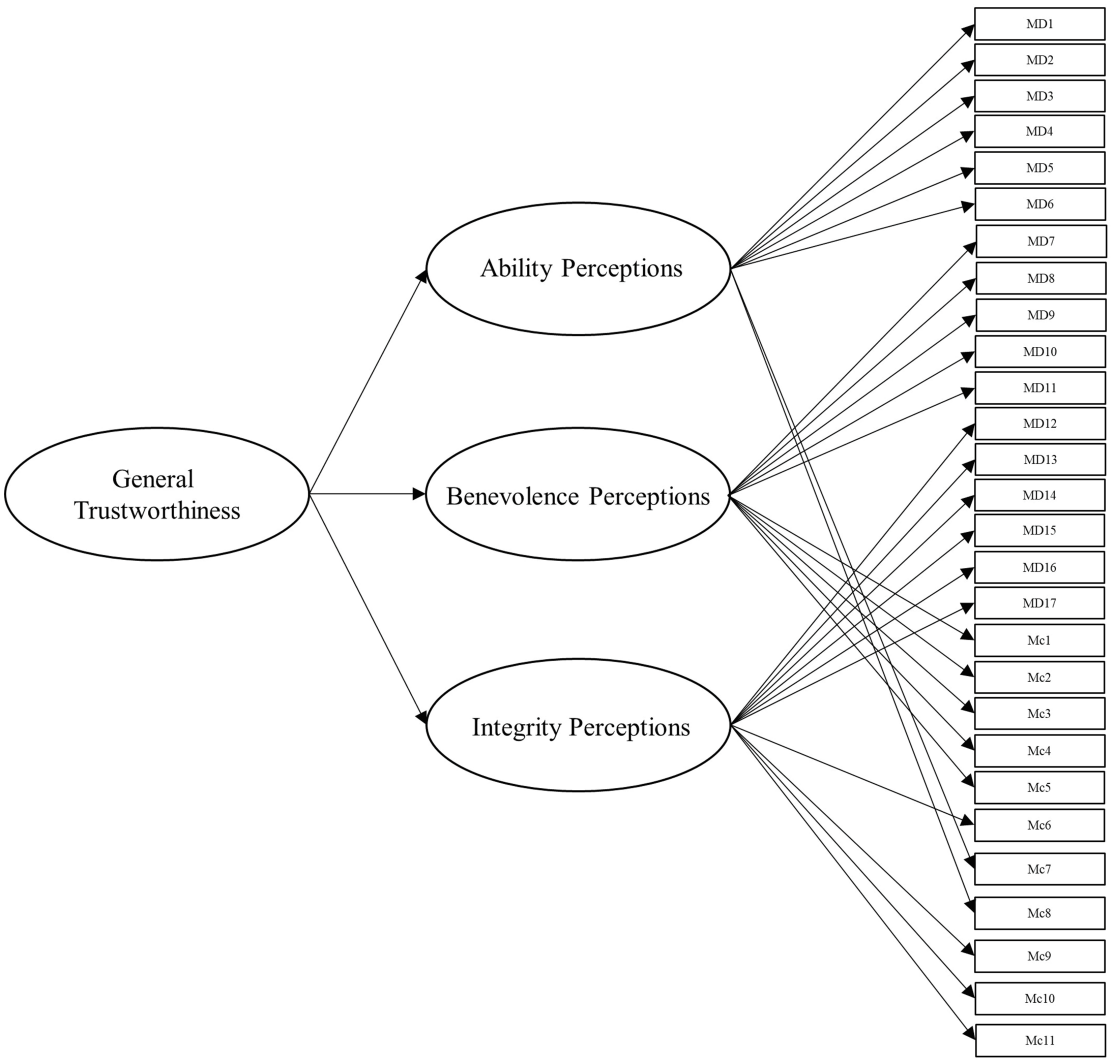
Second-order model with general trustworthiness latent factor. The first-order factors represent ability, benevolence, and integrity. The ability and integrity first-order factors comprise ability and integrity items from Mayer and Davis (1999) as well as cognition-based trust items from McAllister’s (1995) scale (which were delineated by the research team, see Data Analysis) combined. The benevolence latent factor comprises Mayer et al.’s (1995) benevolence items as well as affect-based trust items from McAllister (1995) combined. MD, Mayer and Davis (1999) items. Mc, McAllister (1995) items.

**Figure 6:**
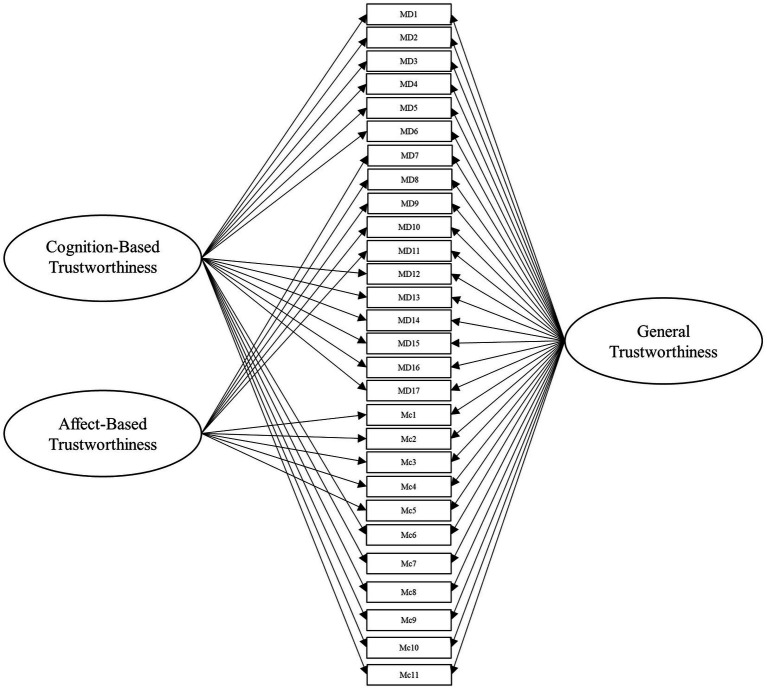
Bifactor model with a general trustworthiness latent factor comprising items from Mayer and Davis (1999) and items from McAllister’s (1995) scales. The grouping factors represent cognition- and affect-based latent factors. The cognition-based trust latent factor comprises ability and integrity items from Mayer and Davis (1999) as well as cognition-based trust items from McAllister (1995). The affect-based trust latent factor comprises Mayer et al.’s (1999) benevolence items as well as affect-based trust items from McAllister (1995) combined. MD, Mayer and Davis (1999) items. Mc, McAllister (1995) items.

**Figure 7:**
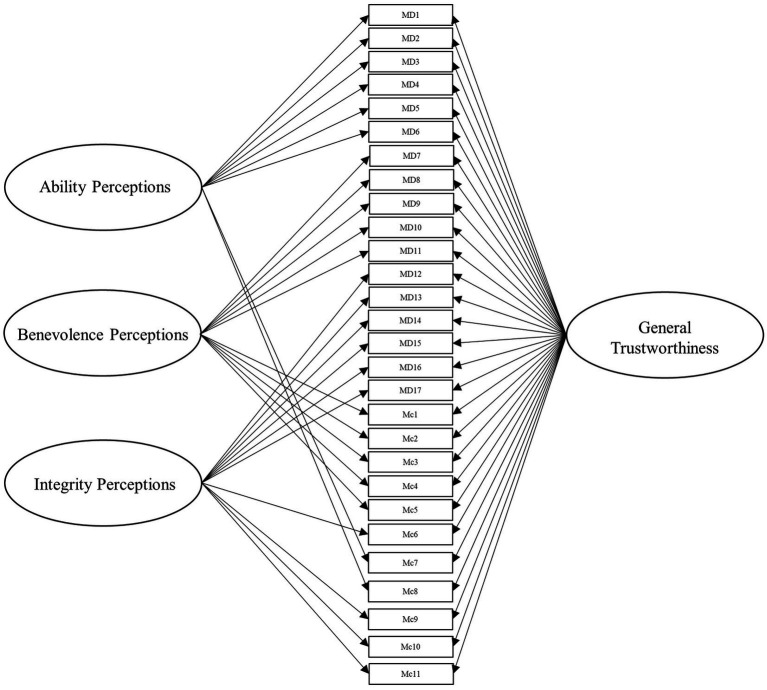
Bifactor model with a general trustworthiness latent factor comprising items from Mayer and Davis (1999) and items from McAllister’s (1995) scales. The grouping factors represent ability, benevolence, and integrity latent factors. The ability and integrity factors comprise ability and integrity items from Mayer and Davis (1999) as well as cognition-based trust items from McAllister’s (1995) scale (which were delineated by the research team, see Data Analysis) combined. The benevolence latent factor comprises Mayer et al.’s (1995) benevolence items as well as affect-based trust items from McAllister (1995) combined. MD, Mayer and Davis (1999) items. Mc, McAllister (1995) items.

To answer this, we leveraged several different specifications for modeling the items from these scales. Beyond the measurement model, the simplest structure to model the data is the unidimensional model, which is depicted in Figure 1. Within this model, all items load onto one general factor (i.e., perceived trustworthiness). Although this model has the benefit of simplicity, it fails to align with our theoretical models of interest (Mayer et al., 1995; McAllister, 1995). Second, we modeled the data according to interpretations (e.g., Dietz and Hartog, 2006; McEvily and Tortoriello, 2011) of McAllister’s (1995) model of trust, with two latent factors comprising cognition- and affect-based trustworthiness, as depicted in Figure 2. To account for the large intercorrelations among the two factors, we could allow the residuals to covary in this model (see DeMars, 2013). Third, we modeled the data according to Mayer et al.’s (1995) three-factor trustworthiness model with three separate latent factors that correspond to ability, benevolence, and integrity, which is illustrated in Figure 3. As with the previous model, we allowed the residuals to covary. Another typical model structure is the second-order (or higher-order) multidimensional model. The higher-order model is typically represented by a second-order factor that directly explains the variation in the first-order latent factors, which comprise variance associated with the items (Watkins and Beaujean, 2014). Thus, the overall factor influences the item indirectly through the first-order factors (Yung et al., 1999; Gignac, 2006). We modeled two second-order latent factor models, one representing interpretations of McAllister’s (1995) model (Figure 4) and one representing Mayer et al.’s (1995) model (Figure 5). In contrast, the bifactor model can model item variance directly from both the general factor and the grouping factors simultaneously (Reise, 2012; DeMars, 2013). This allows researchers to see the direct effects of the grouping factors (or lower-order factors) independent of the general factor. As such, all latent factors are theoretically, and statistically, assumed to be orthogonal to one another (Reise, 2012). As noted by DeMars (2013), the interpretation of the grouping factor scores can be complex for bifactor models. Notably, while the variance contained within each grouping factor is wholly unique to that factor, any shared variance is contained within the general factor. In other words, the grouping factors represent the unique collections of variance that remain after capturing all non-unique variance within the general factor. Additionally, the expected scores from the specific grouping factors are the effects of the grouping factors beyond the effects of the general factor, not the person’s overall standing on the lower-order factor. However, Gignac (2016) demonstrated the higher-order model and the bifactor model are equivalent when the proportionality constraint is tenable (see Yung et al., 1999 for an in-depth explanation and corresponding proofs). We tested the bifactor model using both interpretations of McAllister’s (1995) model and Mayer et al.’s (1995) model as the basis for the grouping factors. These can be seen in Figures 6, 7, respectively. We tested both the second-order latent factor models and the bifactor models and considered the general trustworthiness factor to determine if the bifactor was overfitting.

Additionally, we sought to determine the predictive validity of these measures of trustworthiness based on their factor structure. Previous research has established that trustworthiness predicts a plurality of organizational outcomes. A meta-analysis conducted by Colquitt et al. (2007) showed that—in accordance with Mayer et al.’s (1995) postulates—trustworthiness (i.e., ability, benevolence, and integrity) is related to trust. Additionally, Colquitt et al. (2007) found that trust and its antecedents significantly predict performance, citizenship behaviors, and counterproductive behaviors. Likewise, multiple studies have found that trustworthiness perceptions in one’s direct supervisor are a significant predictor for turnover intentions (e.g., Costigan et al., 2011, 2012). Therefore, we applied the results of our trustworthiness factor analyses to these workplace outcome variables. Specifically, we were interested in the practicality of using the general factor of the best fitting bifactor model to predict criteria and the additional influence of the grouping factors on criteria, if any. Given that these grouping variables would be orthogonal to the general factor (Reise, 2012), their potential predictive influence would raise questions as to what is truly captured by the scale(s) in question. As we do not have any theoretical reason to support the grouping factors either counting for additional variance or not, we considered the following exploratory hypotheses:

*Research Question 2*: Does the general factor of the bifactor model significantly predict workplace outcome criteria, such as trust, performance, organizational citizenship behaviors, counterproductive work behaviors, and turnover intentions?

*Research Question 3*: Do the grouping variables of the bifactor model account for significant variance in criterion variables beyond that of the general factor of trustworthiness?

## Materials and Methods

### Participants

For determining sample size for our analyses, we relied on the rules of thumb described by Comrey and Lee (1992). Based on this, we determined a sample size of approximately 500 would be sufficient for our goals. Respondents completed the trustworthiness and workplace outcomes items on Amazon’s Mechanical Turk (MTurk), a crowdsourcing website where people complete paid online assignments called human intelligence tasks (HITs). We leveraged CloudResearch (formerly TurkPrime, Litman et al., 2017), a platform which interfaces with MTurk for efficient human-subjects social science studies, to post our HITs and track data collection progress. After respondents (known as “workers” on MTurk) enroll in a HIT, they complete the task within a certain time period and are compensated for their work. In the current study, we compensated workers $6.00 USD to complete the survey. All participants were required to be at least 18 years old, located in the United States, proficient in the English language, and currently employed while working a minimum of 30 h per week. We created a HIT that allowed for a maximum of 600 respondents, which was due to concerns with data quality for collection of online samples (see Buchanan, 2000 for a review). A total of 569 participants completed the survey. All respondents were then checked for data quality and indicators of careless responding (see Data Cleaning section below). Following data cleaning, 85 participants were removed, leaving 484 participants used for analysis. The mean age for participants was 35.76 (*SD* = 9.26). A total of 66.32% participants identified as male, 33.05% identified as female, and 0.06% identified as a gender identity not listed on the survey.

### Measures

#### Trustworthiness

Perceived trustworthiness in the participants’ supervisor was measured using 17 items from Mayer and Davis’ (1999) trustworthiness scale, which comprises ability (6 items), benevolence (5 items), and integrity (6 items) factors. We also included McAllister’s (1995) 11-item scale, which assesses cognition- and affect-based trust. As mentioned previously, McAllister’s scale items assess perceptions of a referent’s trust*worthiness* if one leverages the nomenclature Mayer et al. (1995) adopted when explicating the trust process. Cognition-based trustworthiness toward the worker’s supervisor was measured using six items and affect-based trustworthiness toward the worker’s supervisor was measured using five items. Respondents answered items using a seven-point Likert-scale ranging from one (*strongly disagree*) to seven (*strongly agree*). The Cronbach’s alpha estimates from the current study are presented in Table 1.

**Table 1:** Zero-order correlations of study variables.

	1	2	3	4	5	6	7	8	9	10
1. Affect-Based	(0.86)									
2. Cognition-Based	0.49^**^	(0.79)								
3. Ability	0.58^**^	0.61^**^	(0.83)							
4. Benevolence	0.73^**^	0.51^**^	0.64^**^	(0.83)						
5. Integrity	0.50^**^	0.69^**^	0.67^**^	0.59^**^	(0.75)					
6. Trust	0.32^**^	0.58^**^	0.38^**^	0.39^**^	0.56^**^	(0.46)				
7. Performance	0.42^**^	0.74^**^	0.59^**^	0.50^**^	0.67^**^	0.57^**^	(0.77)			
8. OCBs	0.53^**^	0.39^**^	0.40^**^	0.52^**^	0.35^**^	0.22^**^	0.30^**^	(0.81)		
9. CWBs	0.03	−0.32^**^	−0.17^**^	−0.05	−0.32^**^	−0.36^**^	−0.38^**^	0.02	(0.97)	
10. Turnover Int.	−0.10^*^	−0.30^**^	−0.24^**^	−0.17^**^	−0.31^**^	−0.33^**^	−0.32^**^	−0.01	0.56^**^	(0.87)

*N* = 484. Affect-Based, Items from affect-based trust subscale of McAllister (1995); Cognition-Based, Items from cognitive-based trust subscale of McAllister (1995); Ability, Items from ability subscale of Mayer and Davis (1999); Benevolence, Items from benevolence subscale of Mayer and Davis (1999); Integrity, Items from integrity subscale of Mayer and Davis (1999); OCBs, Organizational citizenship behaviors; CWBs, Counterproductive work behaviors; Turnover Int., Turnover intentions.

** *p* < 0.01,

* *p* < 0.05.

#### Trust

We used Mayer and Davis’ scale (Mayer and Davis, 1999) to measure participants’ willingness to be vulnerable with their supervisor, fitting the Mayer et al. (1995) definition of trust. The four items on this measure were assessed using a five-point scale that ranged from one (*strongly disagree*) to five (*strongly agree*). An example is “I would be willing to let my boss have complete control over my future in the company.”

#### Performance

We assessed participants’ ratings of their supervisor’s performance using McAllister’s four-item scale (Mayer et al. 1995). Items were assessed using a seven-point scale that ranged from one (*not at all*) to seven (*entirely*). A sample item is “To what extent are you satisfied with the total contribution made by your supervisor?”

#### Organizational Citizenship Behaviors

We used Kelloway et al.’s scale (Kelloway et al., 2002) to assess participants’ tendency to engage in discretionary behaviors above and beyond their contractual duties that benefit their organization. This was a nine-item measure that included a range of behaviors and asked participants how characteristic each was to how they typically behaved at work. This measure used a five-point Likert rating scale ranging from one (*not at all characteristic*) to five (*very characteristic*). An example is “Volunteering to do things not formally required by the job.”

#### Counterproductive Work Behaviors

We assessed participants’ tendency to engage in intentional behavior that was harmful to their organization’s interests using Kelloway et al.’s scale (Kelloway et al., 2002). This was a 10-item measure that used a five-point scale to rate participants’ frequency in engaging in various behaviors, ranging from one (*never*) to five (*very often*). A sample item is “Covered up your mistakes.”

#### Turnover Intentions

We used three items previously employed by Mitchell et al. (2001) to assess turnover intention. The first item, “Do you intend to leave the organization in the next 12 months?,” used a *Yes*/*No* criterion. The second item, “How strongly do you feel about leaving the organization within the next 12 months?” was assessed using a five-point scale ranging from one (*very strongly*) to five (*not at all*). The final item, “How likely is it that you will leave the organization in the next 12 months?” was assessed using a five-point scale ranging from one (*very likely*) to five (*not at all*).

### Data Cleaning

In order to decrease the likelihood of low-quality responding, we prefaced our study with a paragraph from Zhou and Fishbach (2016), which emphasized the importance of complete, high-quality data for research and that if many participants were to quit midway through the study, our data quality would suffer. Participants were asked to acknowledge this statement and reaffirm their intent to continue with the study. In addition, *post-hoc* metrics employed for data cleaning included checking for missing data and correct responses to attention check items, as well as completion time for survey pages (see Huang et al., 2012). We first removed all participants who had missing data and had failed to correctly answer both attention check items distributed throughout the survey. The attention check items were instruction items, asking participants to select a specific response (e.g., “Please select Strongly Agree for this item”; Huang et al., 2012). We then checked the remaining participants for time spent on each page of the survey, using the criterion proposed by Huang et al. (2012) of an average of 2 s per each item on a survey page to determine careful and attentive responding (e.g., spending less than 20 s on a page with 10 items could be indicative of careless responding). Participants were flagged by page using this criterion and the number of flags for each participant was summed. Participants who had been flagged on more than five pages across the whole battery were considered careless responders and removed. For a copy of the cleaned data used in our analyses, see Data Sheet 1 in the Supplementary Material.

### Analyses

Data were analyzed using the *lavaan* package (Rosseel, 2012) in R (version 4.1.0). For additional analyses specifically used for the bifactor models, we used the *BifactorIndicesCalculator* package (Dueber, 2017). *Post-hoc* power analyses were conducted using the *semPower* package (Moshagen and Erdfelder, 2016). To compensate for the increased variance that occurred from using items from multiple scales in these models, we used the variance standardization method of model fit. We conducted our initial measurement model with 5 factors, one for each trustworthiness facet of the two scales (Model 0). Next, we modeled the unidimensional model, representing one trustworthiness factor for all items and no grouping factors (Model 1, see Figure 1). We subsequently tested a two-factor model representing interpretations of McAllister’s (1995) theoretical model with the ability and integrity items from Mayer and Davis’ (1999) scale loading onto the cognition-based factor and the benevolence items loading onto the affect-based factor (Model 2, see Figure 2).

We then created a three-factor model representing Mayer et al.’s (1995) theoretical model (Model 3, see Figure 3). To our knowledge, few researchers have attempted to delineate McAllister’s (1995) cognition- and affect-based items into ability, benevolence, and integrity (for an exception, see Dietz and Hartog, 2006). Accordingly, the authors (alongside several additional researchers recruited from their lab) independently rated the items on whether they represent ability, benevolence, integrity, or general trustworthiness, based on the theoretical definitions of the constructs. This method was chosen out of a consensus that basing our ratings on well-established theoretical definitions of the constructs would be sufficient for the purpose of this study. Additionally, we sought to constrain the scope of the project, as an exploratory factor analysis would require the collection of an entirely separate sample. We used these ratings (from five researchers in total) to determine which factor to load the cognition-based items for Model 3 and all subsequent models incorporating Mayer and Davis (1999) factors. The affect-based items were theorized to be synonymous with benevolence, so we loaded all affect items from McAllister’s scale onto the benevolence factor. In the next model, we tested a second-order latent factor model with two factors, representing cognition- and affect-based trust (Model 4, see Figure 4). Afterward, we tested our second-order latent factor model with three factors, comprising ability, benevolence, and integrity (Model 5, see Figure 5). To ensure the second-order latent factor was not over-represented by any of the first-order factors, we used effects coding to identify the second-order factors for Models 4 and 5 (Little et al., 2006). Model 6 (see Figure 6) was a bifactor model with one general factor and two grouping factors, representing interpretations of McAllister’s (1995) theoretical model, while Model 7 (see Figure 7) was a bifactor with three grouping factors taken from Mayer et al.’s (1995) theoretical model. Finally, we included a *post-hoc* model based on the results to serve as an additional check of fit. This was a bifactor model treating the five factor variables used in Model 0 as grouping variables alongside a general trustworthiness factor (Model 8).

We chose three changes in fit statistics to determine the relative fit of each model. First, the chi-square (*χ^2^*) fit index was utilized to reveal the degree of fit between nested models, as it has a testable significance assessment (Vandenberg and Lance, 2000). However, the *χ^2^* fit index can be affected by sample size (Shi et al., 2019). Cheung and Rensvold (2002) have suggested using the change in comparative fit index (CFI) and Tucker Lewis index (TLI). Specifically, they recommended a guideline of a change of less than 0.01 for both indices to indicate the adequate invariance assumption. However, there are no steadfast rules for the CFI and TLI measures. As a result, all three measures (Δ*χ^2^*, ΔCFI, and ΔTLI) were used in assessment of the relative fit of the models. Lastly, we extracted the estimated factor scores from the best fitting bifactor model and used the variables to predict the criterion variables of perceived supervisor performance, organizational citizenship behaviors, counterproductive work behaviors, and turnover intentions.

## Results

The reliability estimates and intercorrelations for all variables are illustrated in Table 1. Our final sample provided an *N*:item ratio of approximately 17.29, which is well above the minimum ratio for structural modeling as recommended by Bentler and Chou (1987). *Post-hoc* power analyses calculated using RMSEA values found that all values were sufficiently powered (1 – *β* > 0.99 for all models). First, we conducted CFAs on the trustworthiness scales separately, to ensure the proposed structures fit the data well before combining the scales. The Mayer and Davis (1999) scale had adequate fit, *χ^2^*(116) = 358.80, *p* < 0.001, CFI = 0.93, TLI = 0.91, RMSEA = 0.07, SRMR = 0.05. The McAllister (1995) scale demonstrated poor fit, *χ^2^*(43) = 310.74, *p* < 0.001, CFI = 0.89, TLI = 0.85, RMSEA = 0.11, SRMR = 0.07. Modification indices indicated most of the affect-based items cross loaded onto the cognition-based trustworthiness factor. The last cognition-based item cross-loaded onto the affect-based trustworthiness factor, but it was deemed close enough to the established cutoffs. As such, all items from both scales were incorporated into subsequent analyses.

### Single-Order and Second-Order Models

Next, we fit our models to the data. Fit results of all models are illustrated in Table 2, while the results of the model fit comparisons can be found in Table 3. Model 0, our measurement model, had poor fit. Models 1 through 5, which included all single-order and second-order models, fit the data significantly worse than the measurement model and were subsequently rejected.

**Table 2:** Summary of fit indices for trustworthiness using structural equation modeling.

Model	*χ^2^*	*df*	CFI	TLI	RMSEA	SRMR
Model 0: Measurement Model	1,258.85	340	0.87	0.85	0.07	0.07
Model 1: Unidimensional Model	1,696.13	350	0.80	0.79	0.09	0.07
Model 2: Two-Factor Model (McAllister, 1995)	1,398.54	349	0.85	0.83	0.08	0.07
Model 3: Three-Factor Model (Mayer et al., 1995)	1,361.97	347	0.85	0.84	0.08	0.07
Model 4: Second-Order Factor Model (McAllister)	1,398.54	349	0.85	0.83	0.08	0.07
Model 5: Second-Order Factor Model (Mayer et al.)	1,451.08	348	0.84	0.82	0.08	0.11
Model 6: Bifactor Model (McAllister)	928.95	322	0.91	0.90	0.06	0.05
Model 7: Bifactor Model (Mayer et al.)	892.47	322	0.92	0.90	0.06	0.05
Model 8: Bifactor Model (Measurement)	1,172.81	322	0.88	0.85	0.07	0.06

*N = 484*.

**Table 3:** Summary of model comparisons for trustworthiness factor structure.

Model Comparison	Δ*χ^2^*	ΔCFI	ΔTLI
Model 0 – Model 1 (Measurement vs. Unidimensional)	−437.37	−0.07	−0.06
Model 0 – Model 2 (Measurement vs. Two-Factor)	−139.69	−0.02	−0.02
Model 0 – Model 3 (Measurement vs. Three-Factor)	−103.11	−0.02	−0.01
Model 0 – Model 4 (Measurement vs. Second-Order, Two-Factor)	−139.69	−0.02	−0.02
Model 0 – Model 5 (Measurement vs. Second-Order, Three-Factor)	−108.71	−0.03	−0.03
Model 0 – Model 6 (Measurement vs. Bifactor, Two Grouping)	329.90	0.04	0.05
Model 0 – Model 7 (Measurement vs. Bifactor, Three Grouping)	366.39	0.05	0.05
Model 7 – Model 8 (Bifactor, Three Grouping vs. Bifactor, Five Grouping)	−280.35	−0.04	−0.05

*N = 484. All values of p for chi-square comparisons <0.001*.

### Bifactor Models

Models 6 and 7, the bifactor models, both showed good fit and had improved fit over Model 0. Due to having the same degrees of freedom, Models 6 and 7 could not be compared directly, but Model 7 demonstrated a better chi-squared value and improved CFI. Therefore, Model 7 was retained as the final model. Model 8, an alternative bifactor model with all five factors from both scales (ability, benevolence, integrity, affect-based trustworthiness, and cognition-based trustworthiness), was considered *post-hoc* because the measurement model was the best-fitting model with no higher order latent traits. This model fit the data poorly and significantly worse than Model 7; thus, it was rejected. Based on these analyses, a bifactor model that employs grouping factors based on Mayer et al.’s model of trustworthiness is most representative of the factor structure for these items. Results for this bifactor model with factor loadings are illustrated in Table 4.

**Table 4:** Factor loadings for final confirmatory structural equation of bifactor model.

Item stem	*λ*_Gen_	*λ*_Ability_	*λ*_Benev_	*λ*_Integ_
A1. We have a sharing relationship. We can both freely share our ideas, feelings, and hopes.	0.62^**^	0.00	0.49^**^	0.00
A2. I can talk freely to this individual about difficulties I am having at work and know that (s)he will want to listen.	0.72^**^	0.00	0.22^**^	0.00
A3. We would both feel a sense of loss if one of us was transferred and we could no longer work together.	0.56^**^	0.00	0.48^**^	0.00
A4. If I shared my problems with this person, I know (s)he would respond constructively and caringly.	0.70^**^	0.00	0.24^**^	0.00
A5. I would have to say that we have both made considerable emotional investments in our working relationship.	0.49^**^	0.00	0.64^**^	0.00
C6. This person approaches his/her job with professionalism and dedication.	0.69^**^	0.00	0.00	0.15^**^
C7. Given this person’s track record, I see no reason to doubt his/her competence and preparation for the job.	0.70^**^	0.09	0.00	0.00
C8. I can rely on this person not to make my job more difficult by careless work.	0.62^**^	−0.06	0.00	0.00
C9. Most people, even those who aren’t close friends of this individual, trust and respect him/her as a coworker.	0.66^**^	0.00	0.00	−0.01
C10. Other work associates of mine who must interact with this individual consider him/her to be trustworthy.	0.74^**^	0.00	0.00	0.01
C11. If people knew more about this individual and his/her background, they would be more concerned and monitor his/her performance more closely. (*Reverse-coded*)	0.17^**^	0.00	0.00	0.72^**^
AB1. My boss is very capable of performing his/her job.	0.58^**^	0.24^*^	0.00	0.00
AB2. My boss is known to be successful at the things he/she tries to do.	0.59^**^	0.31^*^	0.00	0.00
AB3. My boss has much knowledge about the work that needs done.	0.58^**^	0.39^*^	0.00	0.00
AB4. I feel very confident about my boss’s skills.	0.68^**^	0.23^*^	0.00	0.00
AB5. My boss has specialized capabilities that can increase our performance.	0.60^**^	0.22^*^	0.00	0.00
AB6. My boss is well qualified.	0.60^**^	0.46^*^	0.00	0.00
B7. My boss is very concerned about my welfare.	0.58^**^	0.00	0.40^**^	0.00
B8. My needs and desires are very important to my boss.	0.62^**^	0.00	0.37^**^	0.00
B9. My boss would not knowingly do anything to hurt me.	0.62^**^	0.00	−0.10^*^	0.00
B10. My boss really looks out for what is important to me.	0.66^**^	0.00	0.38^**^	0.00
B11. My boss will go out of his/her way to help me.	0.58^**^	0.00	0.18^**^	0.00
I12. My boss has a strong sense of justice.	0.44^**^	0.00	0.00	−0.17^*^
I13. I never have to wonder whether my boss will stick to his/her word.	0.61^**^	0.00	0.00	0.08
I14. My boss tries hard to be fair in dealings with others.	0.55^**^	0.00	0.00	0.12^*^
I15. My boss’s actions and behaviors are not very consistent. (*Reverse-coded*)	0.35^**^	0.00	0.00	0.76^**^
I16. I like my boss’s values.	0.65^**^	0.00	0.00	−0.02
I17. Sound principles seem to guide my boss’s behaviors.	0.66^**^	0.00	0.00	−0.06

*N* = 484. *λ*, standardized factor loadings; Gen, General factor from bifactor model; Ability, Ability grouping factor; Benev, Benevolence grouping factor; Integ, Integrity grouping factor; A, items from McAllister’s (1995) scale that correspond to the affect-based trustworthiness factor; C, items from McAllister’s (1995) scale that correspond to the cognition-based trustworthiness factor; AB, items from Mayer and Davis’ (1995) that correspond to the ability factor; B, items from Mayer & Davis’ (1995) that correspond to the benevolence factor; I, items from Mayer and Davis’ (1995) that correspond to the integrity factor.

** *p* < 0.01;

* *p* < 0.05.

We conducted further analysis to ensure Model 7 was not overfitting the data, as this is a notable risk with bifactor models (Bonifay and Cai, 2017). To this end, we used several bifactor indices to test whether the data are “unidimensional enough” to model the data using a one-factor solution (Mansolf and Reise, 2017). As described by Rodriguez et al. (2016), two such indices are the explained common variance (ECV), which is how much common variance is due to the general overall factor, and the percent of uncontaminated correlations (PUC), which is the ratio between the number of unique correlations present in the correlation matrix affected by one factor over the total unique correlations in the matrix. They state that when ECV and PUC values are both elevated, the general trait can be interpreted as similar enough to the unidimensional model, as well as the bias from the grouping factors interpreted as small enough for the structure to be considered essentially unidimensional. Likewise, this suggests that the model is unlikely to be overfitting the data. For Model 7, the observed ECV value was 0.76 while the PUC was 0.69. The ECV for the ability, benevolence, and integrity factors with respect to the general factor were 0.04, 0.10, and 0.08, respectively. Given the results of the bifactor indices, it seems unlikely the model overfit the data. Furthermore, the two trustworthiness latent factors from the unidimensional and bifactor models were practically equivalent (*r* = 0.99, *p* < 0.01). Taken together, these results suggest that modeling the factor structure of the data is similar enough to a unidimensional model and that treating it as such is reasonable in practice.

### Predictive Validity of the Final Model

Regardless of the results for the bifactor indices, we examined the predictive validity of the general factor as well as the grouping factors of Model 7 in relation to established criteria related to trustworthiness. We performed a hierarchical regression with the first step including the general factor and the second step adding the grouping factors to determine if the grouping factors predicted any additional variance after controlling for the general factor. As illustrated in Table 5, the general trustworthiness factor accounted for variance in trust, perceptions of supervisor performance, and organizational citizenship behaviors. Interestingly, the general factor of trustworthiness was not associated with counter-productive work behaviors nor turnover intentions in the initial step. Next, we added the ability, benevolence, and integrity grouping variables to the equation. At least one of the grouping factors accounted for significant variance for each criterion. The ability grouping factor was a significant predictor of only the trust criterion. The benevolence grouping factor was a significant predictor of trust, organizational citizenship behaviors, and turnover intention. Lastly, integrity was a significant predictor of all the criterion except for organizational citizenship behaviors.

**Table 5:** Hierarchical regression analyses for general and grouping factors predicting criteria.

Criterion	Step 1	Step 2			
	Trustworthiness	Trustworthiness	Ability	Benevolence	Integrity
Trust					
*Β*	0.59^**^	0.57^**^	−0.09^**^	0.14^**^	0.48^**^
Total *F*	251.00	136.40			
*R*^2^Δ		0.19^**^			
Total *R*^2^	0.34	0.53			
Supervisor Performance					
*Β*	0.76^**^	0.75^**^	0.00	−0.02	0.29^**^
Total *F*	657.60	242.50			
*R*^2^Δ		0.09^**^			
Total *R*^2^	0.58	0.67			
OCBs					
*Β*	0.48^**^	0.48^**^	−0.07	0.16^**^	−0.08
Total *F*	145.50	45.50			
*R*^2^Δ		0.05^**^			
Total *R*^2^	0.23	0.28			
CWBs					
*Β*	−0.07	−0.04	−0.06	−0.03	−0.66^**^
F	2.08	93.75			
*R*^2^Δ		0.43^**^			
Total *R*^2^	0.00	0.43			
Turnover Intentions					
*Β*	−0.17	−0.14	−0.03	−0.16^**^	−0.48^**^
Total F	13.74	33.14			
*R*^2^Δ		0.19^**^			
Total *R*^2^	0.03	0.22			

*N = 447. β = standardized regression weights*.

** *p < 0.01*.

## Discussion

This study explored the factor structure of trustworthiness with scales from Mayer and Davis (1999) and from McAllister (1995) to determine the most appropriate factor structure of trustworthiness. Interestingly, the measurement model was the best fitting among the single-order theoretical models (i.e., excluding the second-order or bifactor models). The two-factor and three-factor structures based on the models did not fit well, most likely because of the high number of items. After combining all items from both scales, their respective models demonstrated similar fit, both with the single-order and bifactor models. The Mayer et al. (1995) model demonstrated an improved chi-square fit over the McAllister (1995) model (Δ*χ^2^* = 36.57, *p* < 0.001), but this may have been influenced by the sample size, as fitting larger models to even moderate sample sizes can positively bias chi-square results (Shi et al., 2019). Both bifactor models adequately fit the data, while Model 7 (based on Mayer et al.’s, 1995 theoretical model) fit the data best. The ECV and PUC statistics demonstrated Model 7 was sufficiently unidimensional and therefore was likely not overfitting the data. Additionally, some items, like McAllister’s (1995) sixth cognition-based item, loaded more strongly onto the integrity construct than the general factor (see Table 4). Taken together, these results lend support to conceptualizing trustworthiness as an essentially unidimensional construct and employing these scales accordingly. To that end, researchers have some options for applying these items in their investigations of trustworthiness. In the case of factor analysis, one could potentially model these scales without discretizing them into their specific factors, though as demonstrated here, using such a model would likely have poor statistical fit. An alternative would be to create parcels of items (i.e., averages of item groups) based on subdomains of the scales, which can then be applied to produce a unidimensional set of indicators in an SEM measurement model (Little et al., 2013). Either of these methods are likely to result in a relatively negligible loss of variance, while still being representative of the underlying essential unidimensionality of the trustworthiness construct.

However, results from the regression analyses demonstrated each of the grouping factors still accounted for additional variance in at least one criterion after controlling for the general factor. This complicates our findings and raises several questions regarding the grouping factors. Namely, what exactly are these factors in absence of the variance of general trustworthiness? For example, in contrast to our expectations, the general trustworthiness factor did not account for any variance in counterproductive work behaviors. Instead, the integrity grouping factor was the only significant predictor of counterproductive work behaviors. While the general factor’s lack of effect is surprising, integrity’s unique relationship with counterproductive work behaviors has been well-established, particularly within the personnel selection literature (for a review, see Klotz et al., 2013). However, these findings do not specifically comment on the nature of integrity’s relationship with counterproductive work behaviors *independent* of general trustworthiness, as found in this study. Previous research has posited that integrity encapsulates other qualities in a referent such as *fairness*, *justice*, *consistency*, and *promise fulfillment* (Colquitt et al., 2007), all of which might have the capacity to be observed in a referent independent of their general trustworthiness towards the respondent. It is beyond the scope of this paper to determine whether qualities such as these are being encapsulated within the integrity grouping factor orthogonal to general trustworthiness. Given the unique predictive influence of it observed in this study, it remains an important question for future research.

Another unexpected finding was that the ability grouping factor was negatively related to trust in the final regression equation. This not only runs counter to the literature (e.g., Poon, 2013; Frazier et al., 2015) but is counterintuitive: one would expect perceptions of ability to predict an increased willingness to be vulnerable, rather than a decreased willingness. However, this may have been a statistical artifact due to the extraction of the general trustworthiness factor. Likewise, the notably small ECV of the ability grouping factor (0.04), even in comparison to the other grouping factors, suggests that the ability factor contributes comparably little variance to any other factor in the model, which may account for the unusual results. As we have suggested earlier, it is not possible to determine from a bifactor analysis alone what is substantiating the variance of grouping factors after controlling for the general factor (Rodriguez et al., 2016). An alternative possibility for the peculiar statistical findings across the entire study may be that the scales themselves carry deeper psychometric issues, resulting in the violation of proportionality constraints. Specifically, the items do not comparably contribute to the general factor and the grouping factor they mediate, resulting in a notable proportional imbalance and subsequently unusual results.

In summary, we found the model hypothesized by Mayer et al. (1995) fit the data better than a two-factor solution most akin to interpretations of McAllister’s (1995) model. Subsequently, the bifactor model with three grouping factors for ability, benevolence, and integrity, fit the data best overall. Within the bifactor models, the item loadings were generally stronger for the general factor compared to the grouping factors. The results suggest that participants typically respond to ability, benevolence, and integrity items by indicating their general trustworthiness of the referent, not necessarily the referent’s ability, benevolence, and integrity separately. However, these results are muddled, as we also demonstrated the grouping factors still account for additional variance in various criteria frequently considered in trust and trustworthiness literature. This could be attributable to the grouping factors legitimately having variance that may be theoretically and practically relevant, or possibly mere artifacts of underlying psychometric issues with the scales themselves. While it is beyond the scope of the current study, future research should seek to investigate these grouping factors and scales to determine the exact nature of the variance captured by these factors. Whether it be through straightforward unidimensional assessment, item parceling, or another method of model structuring, our results suggest treating trustworthiness as essentially unidimensional is an acceptable method of assessment for researchers who are interested in the general trustworthiness construct.

### Factors of Trustworthiness

Of the first-order models, the factors of ability, benevolence, and integrity fit the data best. It is important to note that none of the three constructs are purely reflective of cognition-based trust nor affect-based trust. McAllister (1995) stated cognition-based trust must be present prior to establishing affect-based trust, indicating a conceptual overlap. The modification indices on the McAllister scale items here indicated the affect-based items cross-loaded onto the cognition-based factor. Similarly, Mayer et al. (1995) hypothesized integrity is the most salient aspect of trustworthiness in burgeoning interactions, and we contend there is a cognition-based aspect to integrity. In contrast to previous research (Gabarro, 1978; Colquitt et al., 2007), the benevolence and integrity constructs demonstrated unexpectedly low correlation. This may have been due to the inclusion of McAllister’s (1995) scale to the model, as the items in the scale demonstrate many cross-loadings between the cognition- and affect-based scales. Instead, we view it more accurate to classify benevolence as the most reflective of affect-based trust within trustworthiness and ability as the most reflective of cognition-based trust within trustworthiness. As for integrity, it may comprise both cognition- and affect-based attributes. Tomlinson et al.’s model (Tomlinson et al., 2020) conceptualizing trustworthiness has similarly identified the unique position of Mayer et al.’s (1995) integrity factor within the broader construct and has discretized its more affect-based trust and more cognition-based trust elements into two factors: values congruence and behavioral integrity, respectively. The current study lends additional support to this factor structure of trustworthiness (and trust by extension) through showing how the Mayer et al. trustworthiness factors map onto McAllister’s (1995) trust factors *via* cross-loading in various types of structural models.

### Practical Implications

Though Mayer et al. (1995) provided a thorough theoretical rationale for conceptualizing trustworthiness as comprising perceptions of ability, benevolence, and integrity, researchers should consider the extent to which empirical evidence supports the three separate factors of trustworthiness (see also Colquitt et al., 2007). Although the three-factor model fit significantly better than the unidimensional model, the intercorrelations between the latent factors were noticeably large. Thus, respondents appeared to report general perceptions of trustworthiness rather than ability, benevolence, and integrity separately. This is likewise supported by the substantially improved fit of the bifactor model. However, the ability, benevolence, and integrity grouping factors accounted for significant additional variance (approximately 5–43%) in criteria after controlling for the general factor, raising further questions about the underlying constructs being assessed. When predicting counterproductive work behaviors, the general factor did not account for any variance in the criterion, but when the grouping factors were added to the equation, namely integrity, the model accounted for an additional 43% of the variance in the criterion. This is especially surprising, considering the results of the bifactors indices suggesting that the model could be considered unidimensional, in practice. While this could be due to integrity representing more than one trustworthiness factor according to recent models (Tomlinson et al., 2020) or merely reflective of an underlying psychometric flaw in the scales, such a large effect size for a grouping factor in a bifactor model demands careful consideration. Assuming this is genuinely reflective of the grouping factor, it would still be from a construct orthogonal to general trustworthiness. Given that these grouping factor patterns appear to be strongly criterion-dependent, researchers may want to focus on these orthogonal grouping factors within the larger trustworthiness structure, as they may be more contextually relevant than the general factor. For example, there is research to suggest that general trustworthiness may be more applicable in cases where the trusting party does not believe vigilance is required, and that individualized assessment of ability, benevolence, and integrity only occur when that belief is challenged (Hendricks et al., 2015). In situations in which there is expected to be some uncertainty across one or more areas of trustworthiness, it may be more prudent to assess trustworthiness as individualized factors, rather than as a general factor. For researchers who wish to individually model the Mayer et al. (1995) factors while considering the essential unidimensionality of the trustworthiness construct, one potential method of partitioning the data is parceling the items by grouping factor (Little et al., 2013). By parceling the data into the ability, benevolence, and integrity constructs, rather than applying the individual items to the model directly, researchers would be able to generate a unidimensional set of indicators, while maintaining the improved model fit of the bifactor.

Beyond these general concerns, there are additional points of consideration based on our findings. The odd results related to the integrity factor also raise additional questions when assessing for that specific construct. The distribution of factor loadings for items assigned to that construct are highly variable, with two items demonstrating a standardized loading greater than 0.70 and several other items displaying non-significant loadings. Despite this, the variances from this grouping variable accounts for a substantial portion of the variance in predicting at least some workplace outcomes. Integrity’s composition of both cognition- and affect-based aspects of trustworthiness (as measured by Mayer et al., 1995), or possibly comprising both value congruence and behavioral integrity (Tomlinson et al., 2020) may be driving this odd assortment of findings. As trustworthiness comprises both cognition- and affect-based aspects, integrity may be the grouping factor most reflective of general trustworthiness, as it entails both cognition and affect. Such nomological similarity might also contribute to the potential psychometric issues with the scales described earlier. For this reason, we would express caution in attempting to assess integrity perceptions within trustworthiness while using either scale, as more work is needed in psychometrically defining and refining the construct.

Additionally, we observed that benevolence and integrity had larger factor loadings with the general trustworthiness latent factor in the unidimensional and bifactor models. These findings align with the original theoretical model from Mayer et al. (1995), in which ability was not only the last factor chronologically to be incorporated into a trustworthiness assessment, but also the most contextually bound of the three. Additionally, it aligns with the emphasis on situation-person perspective advocated by Thielmann and Hilbig (2015). Specifically, they postulated the importance of the situation in which trust occurs. In the current studies, we focused on trustworthiness perceptions towards one’s supervisor. Arguably, a supervisor’s ability is key to a successful career. Although the current study focused on ability-based instances of trust, there are also non-ability trust instances. Classic games such as the Prisoner’s Dilemma (Wedekind and Milinski, 1996) and the Investor/Dictator Game (Berg et al., 1995) are both trust games from game theory which comprise no ability aspect. As such, a general trustworthiness measure may be sufficient, but contexts that involve ability may involve relevant factors outside the general trustworthiness factor alone (see Alarcon et al., 2018). Likewise, researchers can use general measures of trustworthiness to predict other criteria, as the unidimensionality indices of the bifactor model suggest, but should carefully consider when the context or criteria are expected to heavily rely on the individual grouping factors we have extracted in this study.

### Limitations and Future Research

This study has several limitations that should be taken into consideration. One such limitation is that the factor structure of these scales was assessed within the context of organizational trust and trustworthiness, specifically within the employee-supervisor relationship. While findings related to these scales have been generalized to various contexts across the literature (e.g., Jarvenpaa et al., 2000; Lee and See, 2004; Lewicki et al., 2006, etc.), the possibility remains that these findings are only applicable to the assessment of trustworthiness in employee-supervisor relationships. Additionally, while we required that respondents be currently employed, we did not ask them regarding the specific conditions and nature of their employment nor their relationship with their supervisor beyond the specific scales being assessed. Given the variety of hierarchical structures that can occur at both the organizational and employee-supervisor dyad level, this may have influenced respondents’ perceptions towards their supervisor referent, as well as the workplace outcomes of interest. Future research examining organizational trust and trustworthiness perceptions may wish to examine the effect of hierarchy, organizational structure, and situational factors on these variables.

Another limitation was the poor fit of the McAllister (1995) scale, supporting prior critiques of it (see Dietz and Hartog, 2006). Because we were solely interested in the examining the confirmatory factor structure of this scale alongside the items from Mayer and Davis (1999), we decided that collecting an additional sample solely for exploratory factor analysis would be beyond the scope of the study, instead relying on independent ratings from researchers experienced with the theoretical definitions established by Mayer et al. (1995). Indeed, we concluded that several items from the scale could have loaded onto multiple constructs. While we were able to resolve these discrepancies of interpretation upon subsequent discussion and agreement upon the final model structures, this served as an early warning regarding the ambiguity of the scale. It should also be noted that our final interpretations of the McAllister (1995) scale as applied to the Mayer et al. (1995) constructs diverge from other attempts at applying the scale to that model, such as Dietz and Hartog (2006). Upon further analysis of McAllister’s (1995) scale, many items seemed to be double-loaded, and as such we cannot be certain to which aspects of such items the participants are responding. Furthermore, the wordings of several items are phrased such that they can be ambiguously interpreted. For instance, the item “I would have to say that we have both made considerable emotional investments in our working relationship” indicates that both the respondent and his/her supervisor made emotional investments. However, it is unclear how a participant would respond if only s/he made an investment, while the supervisor did not. Similarly, if the supervisor made an investment and the participant did not, it is unclear how they would respond. The above issues extended across the scale, which may have caused the issues in fitting the McAllister scale in the theoretically relevant models. While we were using this scale to assess trustworthiness rather than trust within the context of this study, future research should take careful consideration of the factor structure for the McAllister scale when they seek to apply it in trust research *via* exploratory or confirmatory factor analysis techniques. Alternatively, researchers may wish to update or remake the 27-year-old scale to improve and solidify its psychometric and theoretical validity for modern usage. Given the potential psychometric concerns raised from the results of this study, it may be worthwhile to inquire more deeply into the scales that have been so commonly employed across the trust literature to this day.

On this note, we have followed the speculation of many scientists before us in assuming that McAllister’s (1995) model of cognition- and affect-based trust can also correspond to cognition- and affect-based trustworthiness, respectively (e.g., Dirks and Ferrin, 2002; Colquitt et al., 2007, 2011; Capiola et al., 2020). Indeed, this common assumption has been highlighted explicitly in trust research. In a review of measures of organizational trust, McEvily and Tortoriello (2011) suggested that for those interested in measuring trustworthiness beliefs, McAllister’s (1995) measure is a relevant option as it “emphasizes different characteristics of the trustee” (p. 38). This, coupled with Dietz and Hartog’s (2006) take that McAllister’s (1995) scale assesses mainly beliefs about another’s competence and benevolence, further justifies researchers’ actions on leveraging McAllister’s scale and theoretical approach to measure and characterize both cognition- and affect-based aspects of trustworthiness. However, this may be a problematic assumption, not only from a theoretical perspective, but also based on the psychometric issues that arose here when attempting to incorporate items from this scale into the model. Both Dietz and Hartog (2006) and our own team have recognized that this scale struggles to not only capture the concepts of Mayer et al. (1995) but may not even effectively capture cognition- and affect-based trust. In resolving our discrepancies in rating, we questioned whether several items assessed anything more specific than general trust/trustworthiness. While we eventually reached an agreement for all items, it further highlights the issues arising from the scale, despite its frequent use in trust research. However, Tomlinson et al. (2020) have recently clarified a more accurate integration of these two models that would not conflate trustworthiness (as measured *via* perceptions of ability, behavioral integrity, benevolence, and values congruence) and cognition- and affect-based trust. As such, using McAllister’s (1995) scale is limited in that it conflates trustworthiness and trust, and future work may wish to follow Tomlinson et al.’s (2020) approach to use more conceptually focused scales to assess trust and trustworthiness (see also Gillespie, 2003).

An additional limitation is that we did not include Tomlinson and colleagues’ model (Tomlinson et al., 2014, 2020) in our analyses. As described earlier, Tomlinson and colleagues have discretized Mayer et al.’s (1995) integrity factor into two factors: behavioral integrity and value congruence, which, respectively, are more robustly related to cognition- and affect-based trust. Due to our focus on the original scales from both Mayer et al. (1995) and McAllister (1995), we chose to limit the scope of this study strictly to the models most often discussed in the literature, rather than attempt to fit them to a factor structure that neither of them were designed to capture.^3^ That being said, this newer model could also provide the benefit of resolving questions of directionality across all the constructs of the Mayer et al. (1995) and McAllister models (1995), with Mayer et al.’s constructs feeding into McAllister’s constructs. While the model created by Tomlinson et al. (2020) presents a promising evolution of the theoretical structure of trustworthiness, future research will need to further solidify the assessment and understanding of the newly proposed factors, possibly including new item development and validation, in addition to further validation of its factor structure and predictive validity.

### Conclusion

We found the bifactor model, with a general trustworthiness factor and three orthogonal grouping factors, showed the best model fit for the ability, benevolence, and integrity factors described by Mayer et al. (1995) with items from Mayer and Davis (1999) as well as items from McAllister’s (1995) scale assessing cognition- and affect-based trust. The application of Mayer and Davis’ (1999) scale for assessing trustworthiness is preferred, but given the modeling results of this study, it should be treated as an essentially unidimensional scale. The general factor from the bifactor model consistently showed the strongest relationship with the latent variable derived from a measure of general perceived trustworthiness of a referent (in this case, one’s supervisor). However, the grouping factors explained additional variance after controlling for the general factor from the bifactor model, which raises further questions for the variance captured by the grouping factors of the model, as well as the more general psychometric properties of the scale itself. As such, if researchers are interested in specific relationships between the factors and related criteria, then the theoretical factors of ability, benevolence, and integrity independent of general trustworthiness should be investigated further. Likewise, investigators may wish to probe deeper into the possibility of flaws within the scales themselves, given the concerns raised here. The field of trust and trustworthiness research has rapidly evolved in the past few decades and as such, we must continually reevaluate our methods and models of inquiry as our understanding of these constructs continues to grow and develop.

## Data Availability Statement

The original contributions presented in the study are included in the article/Supplementary Material, further inquiries can be directed to the corresponding author.

## Ethics Statement

This study was reviewed and approved by Air Force Research Laboratory Institutional Review Board for involving human participants. Written informed consent for participation was not required for this study in accordance with the national legislation and the institutional requirements.

## Author Contributions

This project was initially conceived through a collaboration between ML, GA, and AC, with the initial idea proposed by GA. ML created the online study, conducted data collection, and completed data analysis. GA and AC drafted the Introduction and Discussion sections of the manuscript, while ML wrote the Methods and Results. ML led further drafting and editing of the entire manuscript with contributions from both GA and AC. All authors contributed to the article and approved the submitted version.

## Funding

This research was supported, in part, by the Air Force Research Laboratory (contract # FA-8650-16-D-6616) facilitating study design, implementation, data collection, analysis, and write up.

## Author Disclaimer

The views expressed are those of the authors and do not necessarily reflect the official policy or position of the Department of the Air Force, the Department of Defense, or the U.S. government.

## Conflict of Interest

ML is employed at General Dynamics Information Technology, Inc.

The remaining authors declare that the research was conducted in the absence of any commercial or financial relationships that could be construed as a potential conflict of interest.

## Publisher’s Note

All claims expressed in this article are solely those of the authors and do not necessarily represent those of their affiliated organizations, or those of the publisher, the editors and the reviewers. Any product that may be evaluated in this article, or claim that may be made by its manufacturer, is not guaranteed or endorsed by the publisher.

## Footnotes

^1^Indeed, De Jong et al. (2016) constructed their ninth hypothesis by assuming that perceiving the team as “capable and competent” and “genuinely concerned about [the team’s] welfare” would afford cognition- and affect-based trust, respectively (p. 1138). However, through the lens of Mayer et al. (1995), perceptions of these characteristics describe different perceptions of trustworthiness.

^2^For reproductions of Gillespie’s (2003) scale, see Dietz and Hartog (2006) and McEvily and Tortoriello (2011).

^3^To be fair, McAllister et al. (1995) did not delineate cognition- and affect-based antecedents to trust but rather two forms of trust. Over the years, researchers (perhaps due to conceptual overlap or conflation of trust and trustworthiness in McAllister’s, 1995 scale) have made this assumption (e.g., Dirks and Ferrin, 2002; Dietz and Hartog, 2006; Colquitt et al., 2007, 2011; McEvily and Tortoriello, 2011; Capiola et al., 2020).
